# BAFF promotes proliferation of human mesangial cells through interaction with BAFF-R

**DOI:** 10.1186/s12882-015-0064-y

**Published:** 2015-05-15

**Authors:** Nuoyan Zheng, Donxian Wang, Hongyan Ming, Haiqing Zhang, Xueqing Yu

**Affiliations:** Department of Nephrology, The First Affiliated Hospital of Sun Sat-yet University, Guangzhou, China; Translational Medical Center, The First Affiliated Hospital of Sun Sat-yet University, Guangzhou, China; International Travel Health Care Center, Entry & Exit Inspection and Quarantine Bureau of Guangdong Province, Guangdong, China

**Keywords:** BAFF, BAFF-R, Human mesangial cell, Akt

## Abstract

**Background:**

B cell activating factor belonging to the TNF family (BAFF) is vital for B cell survival, proliferation and activation. Evidence indicates that BAFF is systemically or locally increased in glomerulonephritis (e.g. lupus nephritis, IgA nephropathy). However, the effect of BAFF on human mesangial cells is not known.

**Methods:**

The impact of BAFF on the proliferation of a human mesangial cell line *in vitro* was investigated. The expression of BAFF receptor (BAFF-R) and downstream signal transduction were explored. The influence of BAFF on the expression of related genes was also studied.

**Results:**

Our data indicated that BAFF had a proliferative effect on human mesangial cells, as supported by the results of cell proliferation assays and the inhibited expression of the pro-apoptotic gene Bim. BAFF-R was expressed on the cell membrane of human mesangial cells and blockade of BAFF/BAFF-R binding abrogated the proliferative effect of BAFF on human mesangial cells. BAFF stimulation led to rapid phosphorylation of NF-κBp65, Akt and MAPK p38 kinase in human mesangial cells, whereas it had no effect on the expression of NF-κB p100 and phosphorylation of Erk. The phosphorylation of Akt was very sensitive to blockade of BAFF/BAFF-R ligation, although activation of MAPK p38 and NF-κBp65 was not. BAFF treatment resulted in decreased expression of BAFF-R, which implied negative feedback regulation after its binding.

**Conclusions:**

BAFF promoted proliferation of human mesangial cells, which was mediated via BAFF-R. The BAFF/BAFF-R interaction triggered Akt, p65 and p38 activation, with Akt phosphorylation being tightly dependent on BAFF/BAFF-R interaction.

## Background

B cell activating factor belonging to the TNF family (BAFF) is a pivotal factor for B cell survival, proliferation and maturation [1, 2]. In mice, BAFF deficiency leads to an almost complete loss of follicular and marginal zone B lymphocytes [3]. In contrast, mice over-expressing BAFF develop B cell hyperplasia, excessive immunoglobulin production and a glomerular nephritic syndrome [4]. BAFF has three receptors: transmembrane activator and calcium modulator cyclophilin ligand interactor (TACI), B cell maturation antigen (BCMA), and the BAFF receptor (BAFF-R or BR3) [5, 6]. All three receptors are type III single transmembrane receptors, belonging to the tumor necrosis factor (TNF) family of receptors [5]. Unlike BCMA and TACI, BAFF-R binds solely to BAFF and BAFF/BAFF-R signaling is crucial for primary B cell survival, differentiation and homeostasis, as demonstrated by the fact that transitional, follicular and marginal zone B cells are all reduced in BAFF-R mutant mice [7–9]. Upon binding to BAFF-R, BAFF activates protein kinase B (Akt) and extracellular signal-regulated kinase (Erk) in an IκB kinase (IKK)-1-dependent manner in primary B cells, thereby promoting B cell survival [10]. Notably, activation of BAFF-R recruits TNF receptor associated factor 6 (TRAF6) and induces activation of the phosphatidylinositol-3-kinases (PI3K) pathway through Akt and glycogen synthase kinase 3 beta (GSK3β) in malignant B cells [11]. Moreover, mitogen-activated protein kinase (MAPK) p38 signaling is also involved in BAFF/BAFF-R-mediated B cell chemotaxis [12]. BAFF-R is the unique BAFF receptor for activating the non-canonical nuclear factor kappa-light-chain-enhancer of activated B cells (NF-κB) signaling pathway through the processing of p100 into the active p52 unit in B cells [6, 10, 13, 14], although it can also modestly activate the canonical NF-κB signaling pathway [15].

BAFF is mainly produced by immune cells, including monocytes, dendritic cells, neutrophils, B cells and T cells [6]. However, non-hematopoietic cells, including fibroblast-like synoviocytes, osteoclasts, epithelial cells, adipocytes, astrocytes and renal cell carcinoma cells, are also capable of expressing BAFF under certain conditions [6, 16, 17]. In humans, BAFF-R is reported to be widely expressed by all B cell subsets except for plasma cells resident in the bone marrow [18]. The expression/location of BAFF and BAFF-R is closely related to the pathogenesis of nephritis [19]. Mouse models of lupus nephritis demonstrated an elevated serum BAFF level which was positively associated with disease severity [20]. Furthermore, the selective blockade of BAFF prevented the development of nephritis [21]. Evidence of BAFF and BAFF-R expression by human renal cells is scarce in the literature, although a study by Xu *et al.* reported that BAFF and BAFF-R were expressed in the renal tubular epithelial cells of patients with renal allograft rejection [22].

Mesangial cells account for 30–40 % of the total glomerular cell population and play major roles in glomerular mechanical architecture, ultrafiltration, matrix equilibrium, and the biosynthesis of various factors, and they also have immune cell-like functions [23, 24]. The lack of a single specific marker for human glomerular mesangial cells hampers the study of mesangial cells *in vivo*. However, glomerular mesangial cells differentiated into a human mesangial cell line *ex vivo* have demonstrated significant similarities with *in vivo* mesangial cell responses [25–30]. Proliferation of mesangial cells and expansion of the matrix have been demonstrated in many immune-mediated forms of glomerulonephritis including lupus nephritis [31] and IgA nephropathy [32–34]. The effect of BAFF on human mesangial cells has never been elucidated, although it can be secreted by infiltrating inflammatory cells during glomerulonephritis [35].

In this study, we investigated the proliferative effect of BAFF on a human mesangial cell line *in vitro*, as well as BAFF-mediated downstream signaling pathways and gene expression. The expression of BAFF receptors on a human mesangial cell line was also examined and the effect of blocking BAFF/BAFF-R interaction on signal transduction studied.

## Methods

### Reagents

Major reagents included recombinant human BAFF (310–13, Peprotech, Rocky Hill, NJ, USA);BAFF-R antibody for flow cytometry and western blot (14–9117, eBioscience, San Diego, CA, USA); BAFF-R Fc chimera (1162-BR-050, R&D Systems, Minneapolis, MN, USA); phospho-Akt (2965) and Akt antibodies (9272, Cell Signaling Technology, Danvers, MA, USA); phospho-Erk (Thr202/Tyr204) (9106) and Erk antibodies (9102, Cell Signaling Technology); phospho-MAPKp38 (Thr180/Tyr182) (9211) and MAPKp38 antibodies (9212, Cell Signaling Technology); phospho-NF-κB p65 (Ser536) (3033) and NF-κB p65 antibodies (4764, Cell Signaling Technology); NF-κB p100 antibody(BS1247, Bioworld, China); Na^+^/K^+^-ATPase antibody (BS4259, Bioworld); carboxyfluoresceinsuccinimidyl ester (CFSE) (C34554, Life Technologies, Carlsbad, CA, USA); reverse transcription (RR014A) and real-time PCR kits (DRR036A, TAKARA, Shiga, Japan); TRIzol (15596–18) and DNaseI (AM2235, Life Technologies); protein extraction buffer (P0013, Beyotime, China); MTS Proliferation Assay kit (G3582, Promega, Madison, WI, USA); and a membrane protein extraction kit (89842, Pierce, Thermo Scientific, Waltham, MA, USA).

### Ethical statement

This study obtained approval from the ethics review committee of the first affiliated hospital of Sun Yat-sen University, Guangzhou, China. Written informed consent was obtained from donors from whom samples were taken for *in vitro* study.

### Human mesangial cell culture and treatment

The immortalized human mesangial cell (HMC) line was kindly provided by F. X. Huang (Sun Yat-Sen University, Guangzhou, China) [36] and grown in RPMI 1640 supplemented with 10 % (v/v) fetal bovine serum at 37 °C in a 5 % CO_2_ incubator. Equal numbers of mesangial cells were cultured until 50–60 % confluent and then subjected to serum starvation for 4–6 h before treatment. Recombinant human BAFF protein was added to the cell culture at various concentrations (5–100 ng/mL) as specified in the text. In the BAFF/BAFF-R blocking experiment, BAFF-R Fc chimera (500 ng/mL) was administrated 30 min before BAFF treatment.

### Cell proliferation assays for human mesangial cells

After serum starvation, cells were cultured in medium with 2 % FBS containing vehicle (PBS + 0.02%BSA) or variable concentration of BAFF protein for 48 h. Cell proliferation was measured with a MTS Proliferation Assay kit (Promega) based on the reaction of 3-(4,5-dimethylthiazol-2-yl)-5-(3-carboxymethoxyphenyl)-2-(4- sulfophenyl)-2H-tetrazolium (MTS) in metabolically active cells. Total cell numbers were also manually counted and dead cells were excluded with eosin red staining.

For the CFSE assay, the cells were suspended at a concentration of 2 × 10^6^ cells/mL and incubated with 5 μM CFSE for 5 min at 37 °C and then subjected to extensive washing with PBS. Afterwards, the cells were cultured with BAFF as indicated for 48 h then analyzed by flow cytometry. For the time course analysis of cell proliferation, cell proliferation was measured with the MTS Proliferation Assay kit at time points of 0, 6, 12, 24, 36, 48 and 72 h after administration of 20 ng/mL BAFF.

### Analysis of protein expression by western blot and flow cytometry

For signal transduction, serum-starved mesangial cells were subjected to BAFF treatment for 10 min in medium containing 0.5 % serum. Cells were then lysed with protein extraction buffer (20 mM Tris pH7.5, 150 mM NaCl, 1 % Triton X-100, sodium pyrophosphate, β-glycerophosphate, EDTA, Na_3_VO_4_) supplemented with protease inhibitor, centrifuged and total soluble proteins collected in the supernatant. Twenty micrograms of protein were subjected to SDS-PAGE analysis, and transferred to nitrocellulose membranes for 1.5 h at 100 V using a Bio-Rad transblot apparatus (Bio-Rad, Hercules, CA, USA). After protein transfer, the membrane was blocked with 5 % nonfat dry milk powder in PBS. The membrane was sequentially incubated with primary antibodies and horseradish peroxidase-conjugated secondary antibody (Thermo). Signals were visualized with an enhanced chemiluminescent HRP substrate (Pierce).

For detecting expression of BAFF-R on the surface of human mesangial cells, cells were incubated with primary mouse anti-BAFF-R antibody for 30 min in PBS supplemented with 3 % FBS, followed by APC-conjugated anti-mouse secondary antibody. Irrelevant mouse Ig at the same concentration was used as a negative control. Samples were analyzed by flow cytometry (MoFlo, Beckman Coulter Inc., Indianapolis, IN, USA).

For enrichment of the cell membrane fraction, 5 × 10^6^ cells were harvested and processed using a membrane protein extraction kit (Pierce) according to the manufacturer’s instructions. The membrane and cytosolic fractions were concentrated by ultracentrifugation and subjected to western blot analysis. Na^+^/K^+^-ATPase and Erk kinase were employed as control membrane and cytosolic proteins respectively.

### Real-time PCR analysis

To measure the expression of BAFF receptors and other genes in human mesangial cells, RNA was extracted from human mesangial cells for cDNA synthesis and real-time PCR analysis. The cDNA nucleotide sequences were acquired in GenBank: TACI [GenBank: NM_012452.2], BCMA [GenBank:NM_001192.2], BAFF-R [GenBank:NM_052945.3], BAFF [GenBank:NM_001145645.2], transforming growth factor-β1 (TGF-β1) [GenBank:NM_000660.4], vascular endothelial growth factor A (VEGFA) [GenBank:NM_001025366.2], α-smooth muscle actin (α-SMA) [GenBank:NM_001141945.1], tissue inhibitor of metalloproteinase-1 (TIMP-1) [GenBank: NM_003254.2], platelet-derived growth factor beta polypeptide (PDGF-B) [GenBank: NM_002608.2], interferon-gamma-inducible protein 10 (IP-10) [GenBank: NM_001565.3], interlukin-6 (IL-6) [GenBank:NM_000600.3], IL-1β [GenBank:NM_000576.2], B-cell lymphoma 2 (Bcl-2) [GenBank:NM_000633], Bcl-2-interacting mediator of cell death (Bim) [GenBank:NM_001204106.1], B-cell lymphoma-extra-large (Bcl-XL) [GenBank: NM_001191.2]. Primers for each gene were designed using Primer 3.0 software and confirmed against non-redundant sequences in a human cDNA sequence database. The primer sequences are as follows, BAFF-R: Forward 5′CCCTGGACAAGGTCATCATT3′, Reverse 5′TCTTGGTGGTCACCAGTTCA3′; BCMA: Forward 5′GCAGTGCTCCCAAAATGAAT3′, Reverse 5′GTCCCAAACAGGTCCAGAGA3′; TACI: Forward 5′CATCTCCTGAGGGACTGCAT3′, Reverse 5′TGGTACCTTCCCGAGTTGTC3′; BAFF: Forward 5′CGTTCAGGGTCCAGAAGAAA3′, Reverse 5′GTCCCATGGCGTAGGTCTTA3′; TGF-β1: Forward 5′AAGTGGACATCAACGGGTTC3′, Reverse 5′GTCCTTGCGGAAGTCAATGT3′; VEGFA: Forward 5′AAGGAGGAGGGCAGAATCAT3′, Reverse 5′ATCTGCATGGTGATGTTGGA3′; α-SMA: Forward 5′TTCAATGTCCCAGCCATGTA3′, Reverse 5′GAAGGAATAGCCAC; TIMP-1: Forward 5′AATTCCGACCTCGTCATCAG3′, Reverse 5′TGCAGTTTTCCAGCAATGAG3′; IP-10: Forward 5′GCTGTACCTGCATCAGCATT3′, Reverse 5′TTCTTGATGGCCTTCGATT3′; PDGF-B: Forward 5′CTTTAAGAAGGCCACGGTGA3′, Reverse 5′CTAGGCTCCAAGGGTCTCCT3′; IL-6: Forward 5′CCTGATCCAGTTCCTGCAGA3′, Reverse 5′CTACATTTGCCGAAGAGCCC3′; IL-1β: Forward 5′GGTGTTCTCCATGTCCTTTGTA3′, Reverse 5′GCTGTAGAGTGGGCTTATCATC3′; Bcl-2: Forward 5′GCCTTCTTTGAGTTCGGTGG3′, Reverse 5′GAAATCAAACAGAGGCCGCA3′; Bcl-XL: Forward5′AAGAGAACAGGACTGAGGCC3′, Reverse5′TTGCTTTACTGCTGCCATGG3′; Bim: Forward 5′CCCTACAGACAGAGCCACAA3′, Reverse 5′GTCTTCGGCTGCTTGGTAAT3′.

### Statistical analysis

The results are expressed as the mean ± standard deviation. Statistical analysis of differences between groups was performed using an unpaired Student’s *t*-test. *P*-values less than 0.05 were considered statistically significant.

## Results

### BAFF stimulates human mesangial cell proliferation

**Fig. 1:**
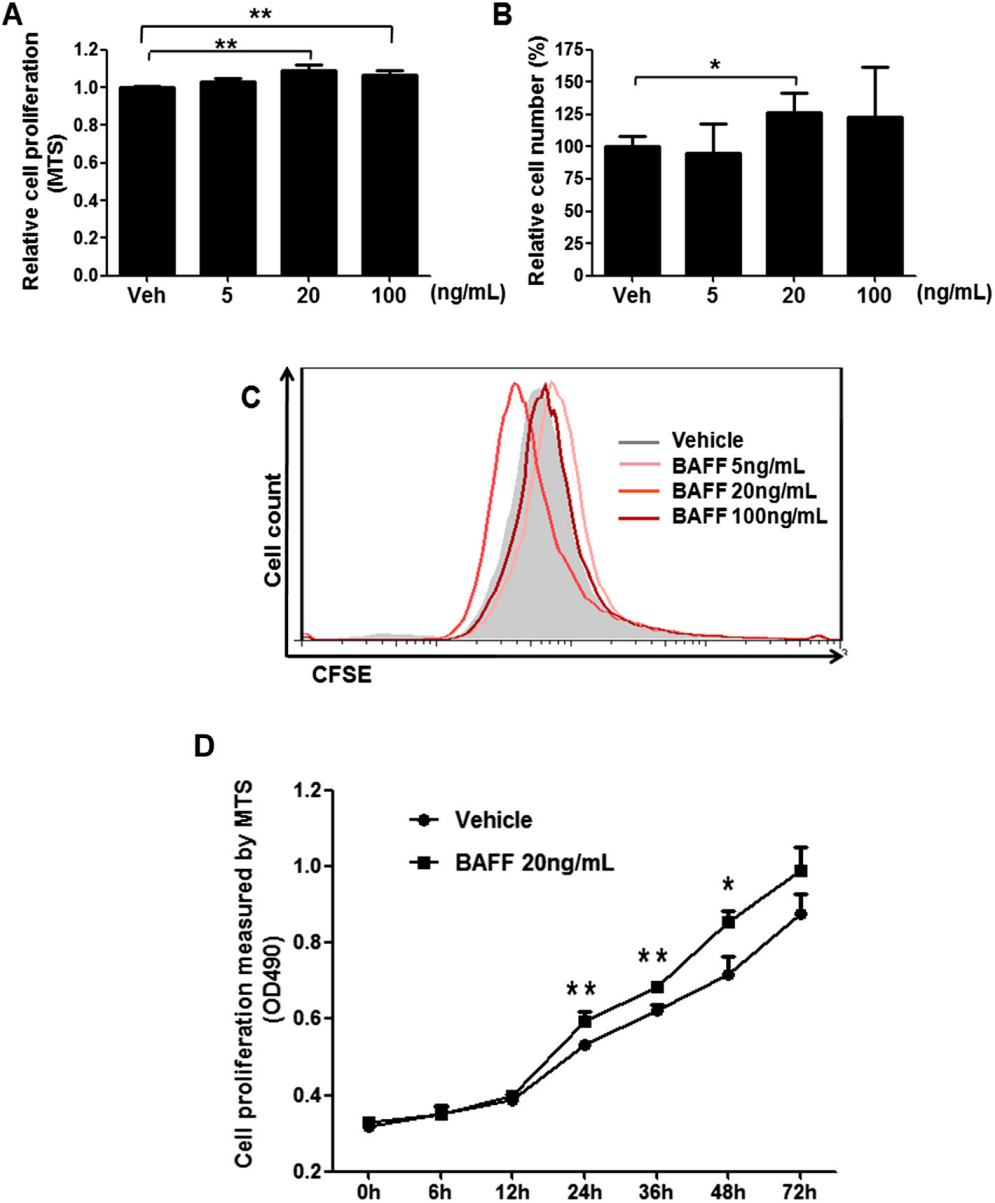
BAFF promotes proliferation of human mesangial cells *in vitro*. Equal numbers of human mesangial cell were subjected to serum starvation for 6 h and then treated with vehicle or BAFF (5, 20, 100 ng/mL) in medium containing 2 % serum medium for 48 h. Cell proliferation was measured with MTS cell proliferation kit (**a**) and alive cells were manually counted (**b**). For CFSE labeling, cells were incubated with CFSE before culture. The proliferation of cells is indicated by the decline of DNA-incorporated CFSE fluorescence (**c**). For the time course assay of proliferation, BAFF (20 ng/mL) was added into the cell culture after serum starvation. Cell proliferation was measured at 490 nm following the instructions of the MTS cell proliferation kit (**d**). Each experiment was repeated independently at least four times and statistical significance is indicated, *p < 0.05, **p < 0.01

To investigate whether BAFF promotes proliferation of human mesangial cells, we cultured a human mesangial cell line *in vitro* with either vehicle or BAFF (5, 20, 100 ng/mL) for 48 h. Based on the result of MTS assays, BAFF significantly accelerated the growth of human mesangial cells at concentrations of 20 and 100 ng/mL (Fig. 1A). Actual cell numbers were manually counted by microscopy with exclusion of positive eosin red-staining cells. The results demonstrated that human mesangial cells significantly responded to BAFF treatment at a concentration of 20 ng/mL, with a 26 % increase in cell numbers compared with the vehicle control. Notably, other dosages had no significant effect (Fig. 1B). Detection of apoptosis by staining with annexin-V and propidium iodide was also conducted in human mesangial cells after 48 h of culture, and less than 3 % apoptosis was observed in all tested groups (data not shown).

When using the DNA-labeling fluorescent dye CFSE as an indicator of cell proliferation, we found that BAFF effectively increased the proliferation of mesangial cells at a concentration of 20 ng/mL (Fig. 1C). Furthermore, we found that BAFF (20 ng/mL) also enhanced the proliferation of human mesangial cells at time points of 24 h (11.8 % increase), 32 h (9.9 % increase), and 48 h (19.2 % increase), and that this effect became less significant at 72 h (Fig. 1D).

### BAFF binding to human mesangial cells triggers signal transduction

**Fig. 2:**
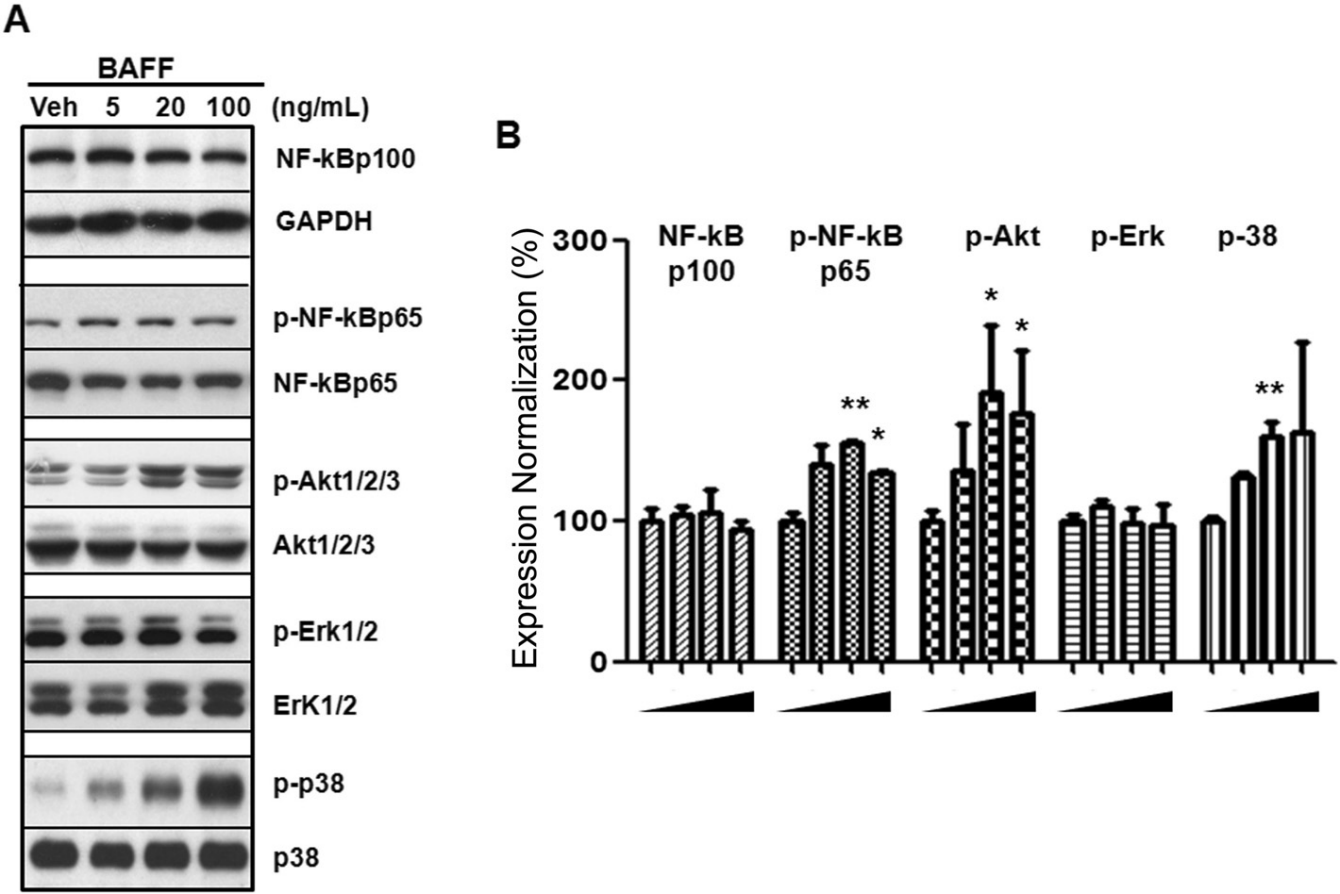
BAFF signal transduction in human mesangial cells. Human mesangial cells were subjected to serum starvation for 4 h, and then BAFF treatment (Veh, 5, 20, 100 ng/mL, indicated with the symbol () for 10 min. The harvested cells were lysed for western blot, with antibodies used as indicated (**a**). **b** This experiment was repeated independently at least three times and expression ratios of target proteins were normalized to GAPDH or the unphosphorylated form of the protein. Specifically, NF-κBp100: NF-κBp100/GAPDH; p-NF-κBp65: p-NF-κBp65/NF-κBp65; p-Akt: p-Akt/Akt; p-Erk: p-Erk/Erk; p-p38: p-p38/p38. Statistical significance is indicated as *p < 0.05, **p < 0.01 as compared with the vehicle group

To further elucidate the mechanism by which BAFF affected the proliferation of mesangial cells, the signaling pathways downstream of BAFF were investigated by western blot. We found that BAFF stimulation led to the rapid phosphorylation of NF-κB p65, Akt1/2/3 and MAPK p38 within 10 min, whereas phosphorylation of Erk1/2 and expression of NF-κB p100 were not up-regulated by BAFF treatment (Fig. 2A). BAFF (20 ng/mL) stimulated the phosphorylation of p65, Akt and p38 1.55-, 1.90- and 1.60-fold of vehicle controls, respectively (Fig. 2B). Consistent with the mesangial cell proliferation profile, the phosphorylation of p65, Akt and p38 protein was most significant at a BAFF concentration of 20 ng/mL, whereas higher or lower dosages were less effective.

### BAFF-R is expressed on the surface of human mesangial cells

**Fig. 3:**
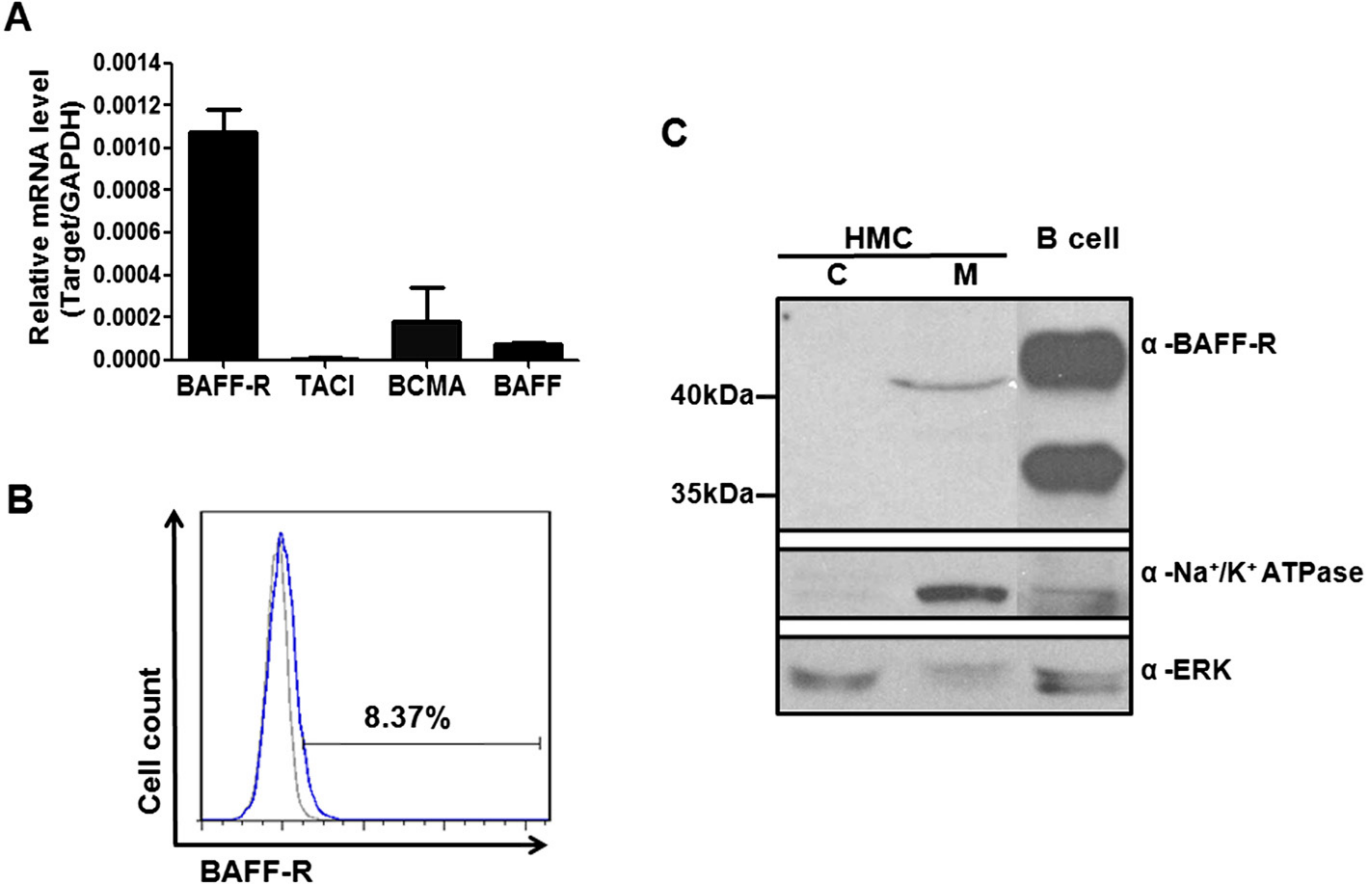
The expression of BAFF receptors by human mesangial cells. **a** Total RNA was extracted from human mesangial cells and analyzed for expression of BAFF-R, TACI, BCMA and BAFF, normalized to GAPDH expression, by real-time PCR. **b** The presence of BAFF-R on the surface of human mesangial cells was detected with BAFF-R antibody by flow cytometry. Background signal is presented as a gray line, and BAFF-R signal is presented as a blue line. **c** Cytosolic protein fraction (indicated as c) and membrane protein fraction (indicated as m) were prepared from human mesangial cells and subjected to western blot analysis. Whole protein lysate of activated human CD19^+^ B cells was loaded as a positive control for expression of BAFF-R. Na^+^/K^+^-ATPase and Erk kinase represented membrane and cytosolic proteins respectively

BAFF has three receptors: BAFF-R, TACI and BCMA. However, evidence of their expression on the surface of human mesangial cells is lacking. We analyzed the mRNA levels of BAFF-R, TACI, BCMA and BAFF in a human mesangial cell line by real-time PCR. The results demonstrated that BAFF-R gene expression was present in human mesangial cells, while the mRNA levels of BAFF, BCMA and TACI were very low (Fig. 3A). The expression of BAFF-R by human mesangial cells was confirmed by flow cytometry, with 8.4 % of human mesangial cells having positive surface staining for BAFF-R as compared with the Ig control (Fig. 3B). Additionally, cell fractions of membrane and cytosolic proteins were prepared from human mesangial cells and subjected to western blot. The results showed that a clear band corresponding in size to BAFF-R protein was detected in the membrane fraction of human mesangial cell, whereas it was not detected in the cytosolic fraction (Fig. 3C). Collectively, these results demonstrated that BAFF-R was expressed on the surface of human mesangial cells, although the abundance was not high.

### BAFF/BAFF-R interaction is essential for the proliferative effect of BAFF on human mesangial cell

**Fig. 4:**
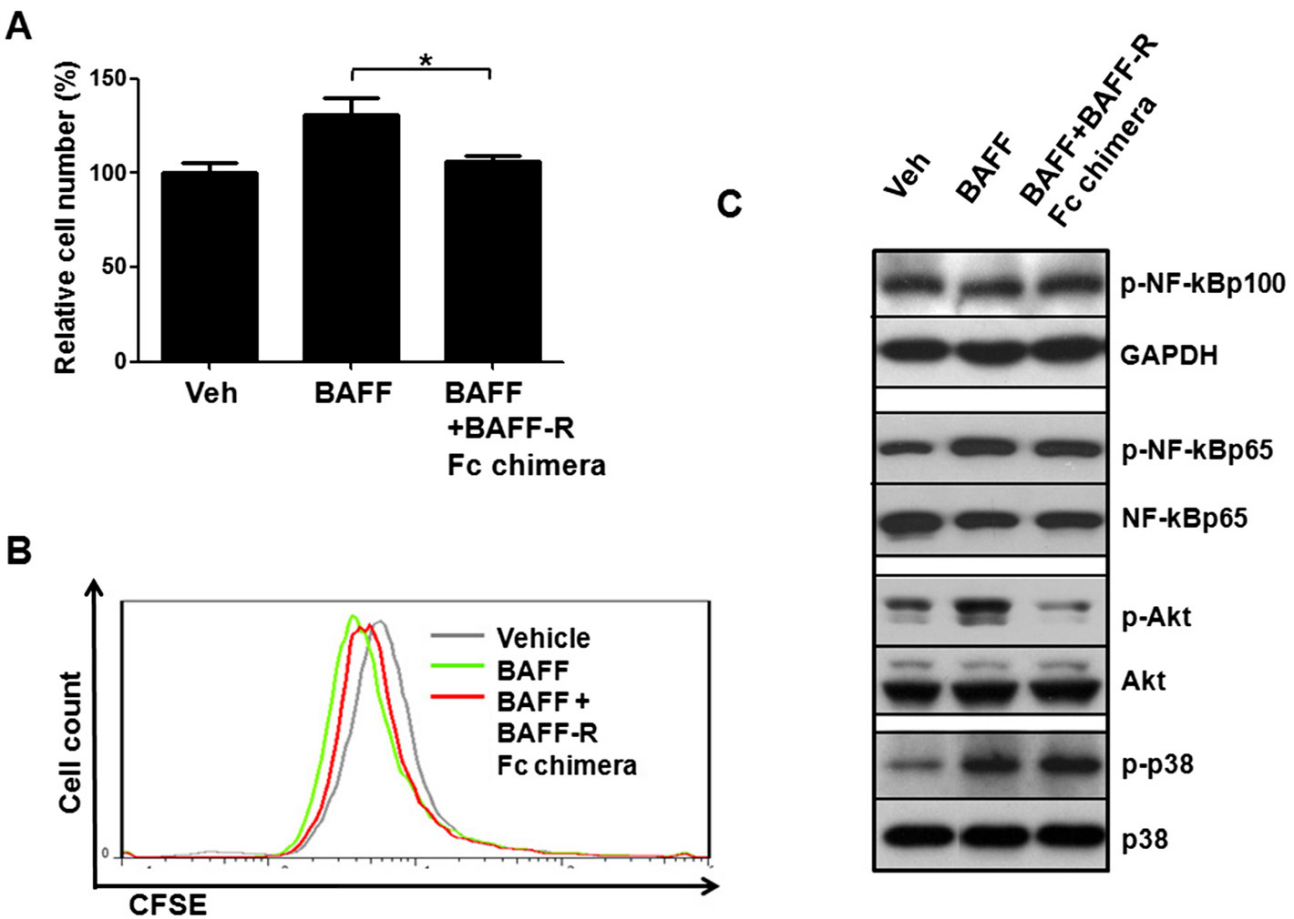
BAFF-R Fc chimera inhibits BAFF-mediated proliferation of human mesangial cells *in vitro*. Human mesangial cells were sequentially subjected to serum starvation for 4–6 h, BAFF-R Fc chimera (500 ng/mL) incubation for 30 min, and finally BAFF treatment (20 ng/mL) in 2 % serum-medium for 48 h (**a**, **b**) or 0.5 % serum-medium for 10 min (**c**). **a** The number of living cells was counted manually. **b** The proliferation of cells is indicated by a decline in DNA-incorporated CFSE fluorescence. **c** Protein from treated cells was analyzed for expression of target proteins by western blot

To analyze whether the proliferative impact of BAFF on human mesangial cells was due to the interaction of BAFF and BAFF-R, we used a BAFF-R Fc chimera protein to compete for the binding of BAFF. In the presence of BAFF-R Fc chimera protein, we found the proliferative response of human mesangial cells after exogenous BAFF protein stimulation diminished (Fig. 4A). CFSE-labeling experiments also showed that proliferation induced by BAFF (20 ng/mL) was attenuated when BAFF-R Fc chimera (500 ng/mL) competed with the cognate membrane-associated BAFF-R for binding to BAFF (Fig. 4B). Furthermore, we found that the BAFF-R Fc chimera inhibited the phosphorylation of Akt, whereas the phosphorylation of NF-κB p65 and MAPK p38 induced by BAFF were less affected by the BAFF-R Fc chimera (Fig. 4C).

### The impact of BAFF on the expression of cytokines and growth factors by human mesangial cells

**Fig. 5:**
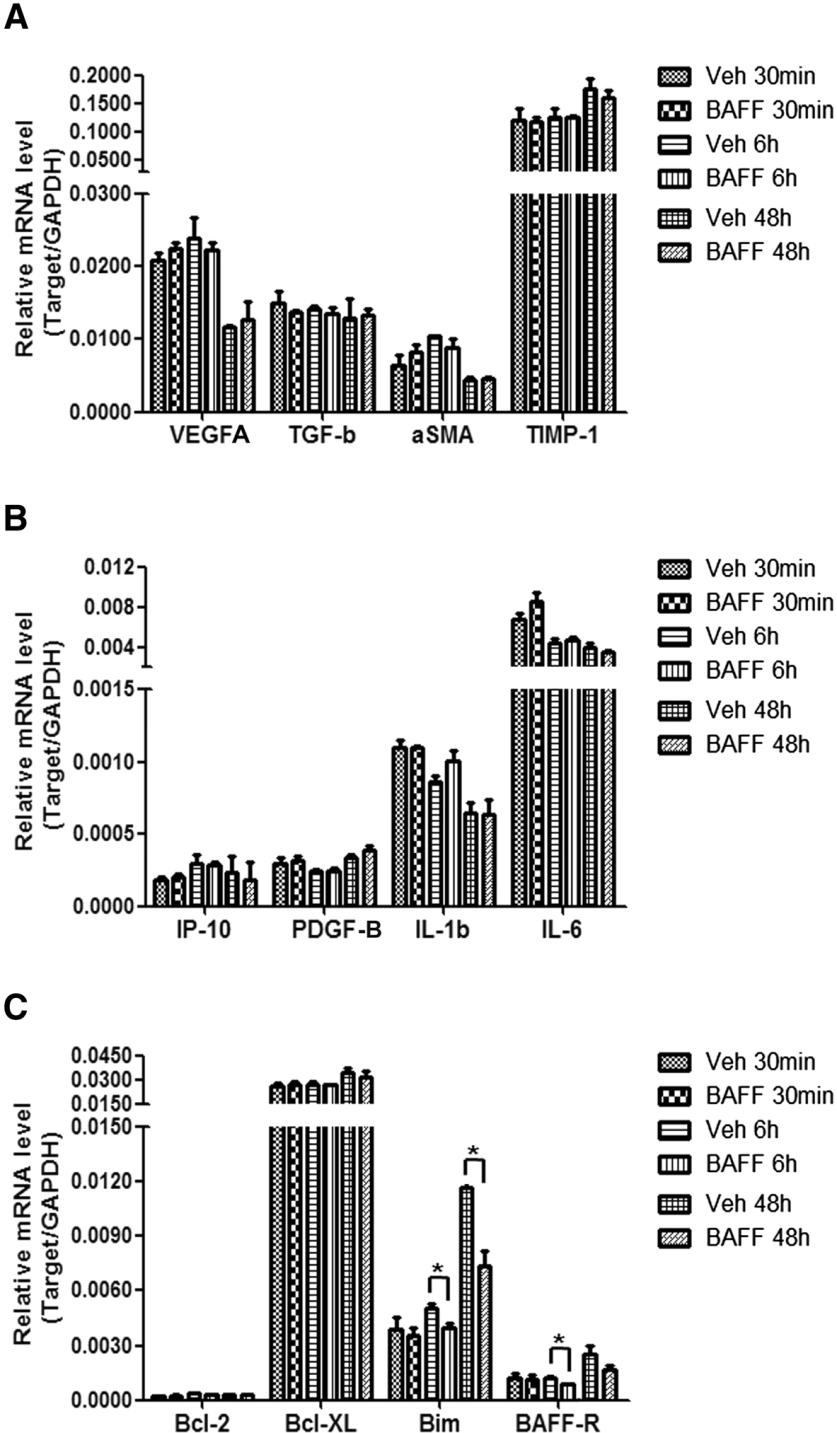
Regulation of gene expression by BAFF in human mesangial cells. Human mesangial cells were subjected to serum starvation for 6 h and then BAFF treatment for 30 min, 6 h or 48 h. The mRNA levels of VEGFA, PDGF-B, TGF-β1, IL-6, IL-1β, IP-10, α-SMA, TIMP-1, Bcl-2, Bcl-XL, Bim and BAFF-R were measured by real-time PCR. This experiment was repeated independently at least three times and representative data are shown. *p < 0.05

To test whether BAFF influences the expression of growth factors, proinflammatory factors, chemokines or bioactive molecules by human mesangial cells in addition to its proliferative effect, we analyzed the expression of VEGFA, PDGF-B, TGF-β1, IL-6, IL-1β, IP-10, α-SMA and TIMP-1 after BAFF treatment for 30 min, 6 h and 48 h. However, the results showed that BAFF had no significant effect on the expression of these molecules (Fig. 5A, B). The mRNA levels of collagen IV, iNOS, fibronectin, MMP2, and MMP9 were very low as detected by real-time PCR, with or without BAFF treatment (data not shown). Additionally, the expression of the apoptosis-related genes Bcl-2, Bcl-XL, and Bim was determined, and the result indicated that the expression of the pro-apoptotic gene Bim was significantly decreased after 6 h (21.3 % reduction) and 48 h (36.8 % reduction) of BAFF treatment. Furthermore, BAFF stimulation decreased BAFF-R expression after 6 h (28.9 % reduction) and 48 h (33.6 % reduction) in human mesangial cells (Fig. 5C).

## Discussion

Over-expression of BAFF has been convincingly proven to cause autoimmune disease and glomerular nephritic syndrome [20, 37]. Many studies found the development of immune-mediated glomerulonephritis was mainly due to the deposition of immune-complexes in the kidney, a consequence of increased binding of BAFF to its receptors and stimulation of B cell function [4, 38]. Additionally, the local concentration of BAFF in the kidney is increased several fold in pathological situations, as previously shown in lupus nephritis [35, 39] and IgA nephropathy [40]. Hence, accumulated BAFF in renal tissue may be an important inflammatory factor mediating renal pathology. Renal BAFF protein could be released by infiltrating immune cells [39] or derived from the peripheral circulation [41], changing the growth factor milieu surrounding resident mesangial cells, in which endogenous BAFF expressed by mesangial cellsis very low as indicated by our data. Whether BAFF influences resident renal mesangial cells was previously unclear. Our results indicated that BAFF retained a proliferative effect on an immortalized human mesangial cell line through interaction with BAFF-R, which suggests that intrinsic renal cells are also very likely responsive to local BAFF protein. We found that BAFF-R was expressed on the surface of a human mesangial cell line *in vitro*. Furthermore, BAFF treatment suppressed subsequent BAFF-R expression, which suggests a negative feedback mechanism. *In vivo* data from lupus nephritis demonstrated that expression of BAFF-R was not detected in innate glomerular tissue [22]. However, the *in vivo* expression profile of BAFF-R and other BAFF receptors in glomerulonephritis (e.g. IgA nephropathy) still requires careful characterization.

The downstream signal transduction of BAFF in B cells passes through several pathways including PI3K-Akt, non-canonical and canonical NF-κB signaling pathways*.* BAFF/BAFF-R interaction preferentially induces alternative NF-κB pathway activation in B cells [14, 42], although classical NF-κB signaling activation is also partially dependent on BAFF-R [14]. In our study, modest activation of the canonical NF-κB signaling pathway was induced by BAFF as indicated by p65 phosphorylation. Patke *et al.* reported that BAFF induced Akt activation in B cells within 10 min and this event was mediated by PI3K activation [43]. We also found BAFF-induced Akt phosphorylation within 10 min in human mesangial cells. Although the MAPK and canonical NF-κB signaling pathways were also activated by BAFF in our study, Akt phosphorylation may play a major role in BAFF/BAFF-R signaling transduction given that BAFF-Fc chimera only significantly inhibited phosphorylation of Akt, but not NF-κB p65 or p38. Over-expression of BCMA activates NF-κB, Elk-1, the c-Jun and p38 in HEK293 cells [44]. Thus, it was worth investigating whether BAFF bound to BCMA and induced downstream NF-κB and p38 activation, considering that a trace amount of BCMA mRNA was detected in human mesangial cells in our study. Although sustained Erk activation is observed in BAFF during B cell death [45], the activation of Erk induced by BAFF in mesangial cells was not significant. The relatively high background of Erk phosphorylation during mesangial cell growth made it difficult to verify any changes.

Growth factors, cytokines and functional molecules are critically important for human mesangial cells to carry out their biological functions. However, the expression of the molecules we examined (VEGFA, PDGF-B,TGF-β1, IL-6, IL-1β, IP-10, α-SMA, TIMP-1) was not influenced by BAFF stimulation. However, notably, the expression of the pro-apoptotic molecule Bim was significantly inhibited in the presence of BAFF for 6 h or 48 h. Bim has been reported to be negatively regulated by Akt-phosphorylation in the process of apoptosis [46], which is in accord with our data. These data suggest that BAFF mainly regulates the proliferation and survival of human mesangial cells, rather than their innate biological function. Still, increased numbers of mesangial cells will contribute to the expansion and altered function of the mesangium, with subsequent consequences.

## Conclusions

Here we found that BAFF exerted a proliferative effect on human mesangial cells *in vitro* through interaction with its unique receptor BAFF-R, which was expressed on the surface of human mesangial cells. Downstream signaling of BAFF/BAFF-R interaction mainly resulted in Akt activation. The proliferative influence of BAFF on human mesangial cells suggests that BAFF may contribute to the pathogenesis of glomerulonephritis.

## Abbreviations

Akt: Protein kinase B
BAFF: B cell activating factor
BAFF-R: BAFF receptor
Bcl-2: B-cell lymphoma 2
Bcl-XL: B-cell lymphoma-extra large
BCMA: B cell maturation antigen
Bim: Bcl-2-interacting mediator of cell death
Erk: Extracellular signal-regulated kinase
GSK3β: Glycogen synthase kinase 3 beta
IgAN: IgA nephropathy
IL-1β: Interleukin-1β
IL-6: Interleukin-6
IKK: IkB kinase
IP-10: Interferon-gamma-inducible protein 10
MAPKp38: Mitogen-activated protein kinases p38
NF-κB: Nuclear factor kappa-light-chain-enhancer of activated B cells
PBS: Phosphate-buffered saline
PDGF-B: Platelet-derived growth factor beta polypeptide
SMA: α-smooth muscle actin
TACI: Transmembrane activator and calcium modulator cyclophilin ligand interactor
TGF-β1: Transforming growth factor-β1
TIMP-1: Tissue inhibitor of metalloproteinase-1
TRAF6: TNF receptor associated factor 6
VEGFA: Vascular endothelial growth factor A
